# Extracellular vesicles: biological mechanisms and emerging therapeutic opportunities in neurodegenerative diseases

**DOI:** 10.1186/s40035-024-00453-6

**Published:** 2024-12-06

**Authors:** Ling Wang, Xiaoyan Zhang, Ziyi Yang, Binquan Wang, Hongyang Gong, Ke Zhang, Yi Lin, Mingkuan Sun

**Affiliations:** The Key Laboratory of Modern Toxicology, Ministry of Education, School of Public Health, Nanjing Medical University, Nanjing, 211166 China

**Keywords:** Extracellular vesicle, Neurodegenerative disease, Biomarker, Therapeutic strategy

## Abstract

Extracellular vesicles (EVs) are membrane vesicles originating from different cells within the brain. The pathophysiological role of EVs in neurodegenerative diseases is progressively acknowledged. This field has advanced from basic biological research to essential clinical significance. The capacity to selectively enrich specific subsets of EVs from biofluids via distinctive surface markers has opened new avenues for molecular understandings across various tissues and organs, notably in the brain. In recent years, brain-derived EVs have been extensively investigated as biomarkers, therapeutic targets, and drug-delivery vehicles for neurodegenerative diseases. This review provides a brief overview of the characteristics and physiological functions of the various classes of EVs, focusing on the biological mechanisms by which various types of brain-derived EVs mediate the occurrence and development of neurodegenerative diseases. Concurrently, novel therapeutic approaches and challenges for the use of EVs as delivery vehicles are delineated.

## Introduction

Neurodegenerative diseases (NDDs) are a category of neurological diseases that affect millions worldwide by causing progressive neuronal death in either the central or the peripheral nervous system. Disruption of neural network structure and function and neuronal loss impair core communication circuits, leading to impairments in cognition and motor function [1]. Despite advancement in the understanding of the pathogenesis of neurodegenerative diseases, the absence of biomarkers impedes the differentiation of disease subtypes in early-stage patients (when it is still possible to maintain acceptable cognitive abilities) and hampers disease progression monitoring. Current studies emphasize that cells can transmit disease-causing molecules to both neighboring and distant cells through extracellular vesicles (EVs), which have emerged as a critical factor in disease progression [2, 3].

EVs are membranous vesicles secreted by cells into the extracellular space, playing a crucial role in both local and long-distance intercellular communication [4]. EVs with cargoes of proteins, lipids, metabolites and nucleic acids, have diverse systemic effects, influencing a wide range of pathological and physiological processes. This has sparked growing interest in EVs as non-invasive biomarkers for diagnosing and prognosticating diseases [5–8]. Current studies are exploring inhibitors to hinder the release or uptake of EVs, aiming to impede disease progression. Recent studies on EVs emphasize their potential as drug-delivery vehicles that encapsulate small molecules, proteins, and small interfering RNAs to achieve precise targeting, thus advancing broad implementation of EV therapies in cancer, cardiovascular, and neurological conditions.

It has been observed that neurons, microglia, astrocytes, and oligodendrocytes in the central nervous system (CNS), as well as peripheral nervous system cells, release EVs into blood circulation. EVs are readily accessible and isolatable from most biological fluids, rendering them appealing and feasible targets for biomarker identification. Moreover, EVs serve as indicators reflecting the pathophysiological condition of the parent cells [9–11]. In addition, EVs are capable of traversing the blood–brain barrier (BBB) directly [12–14], offering the potential for treating neurological disorders. In this review, we provide an overview of the physiological and pathological roles of EVs in NDDs. The emerging therapeutic potential of EVs as brain biomarkers and drug delivery vehicles is discussed alongside critical challenges and future prospects in this field.

## EV classification and neurobiological functions

### Classification and biogenesis

**Fig. 1:**
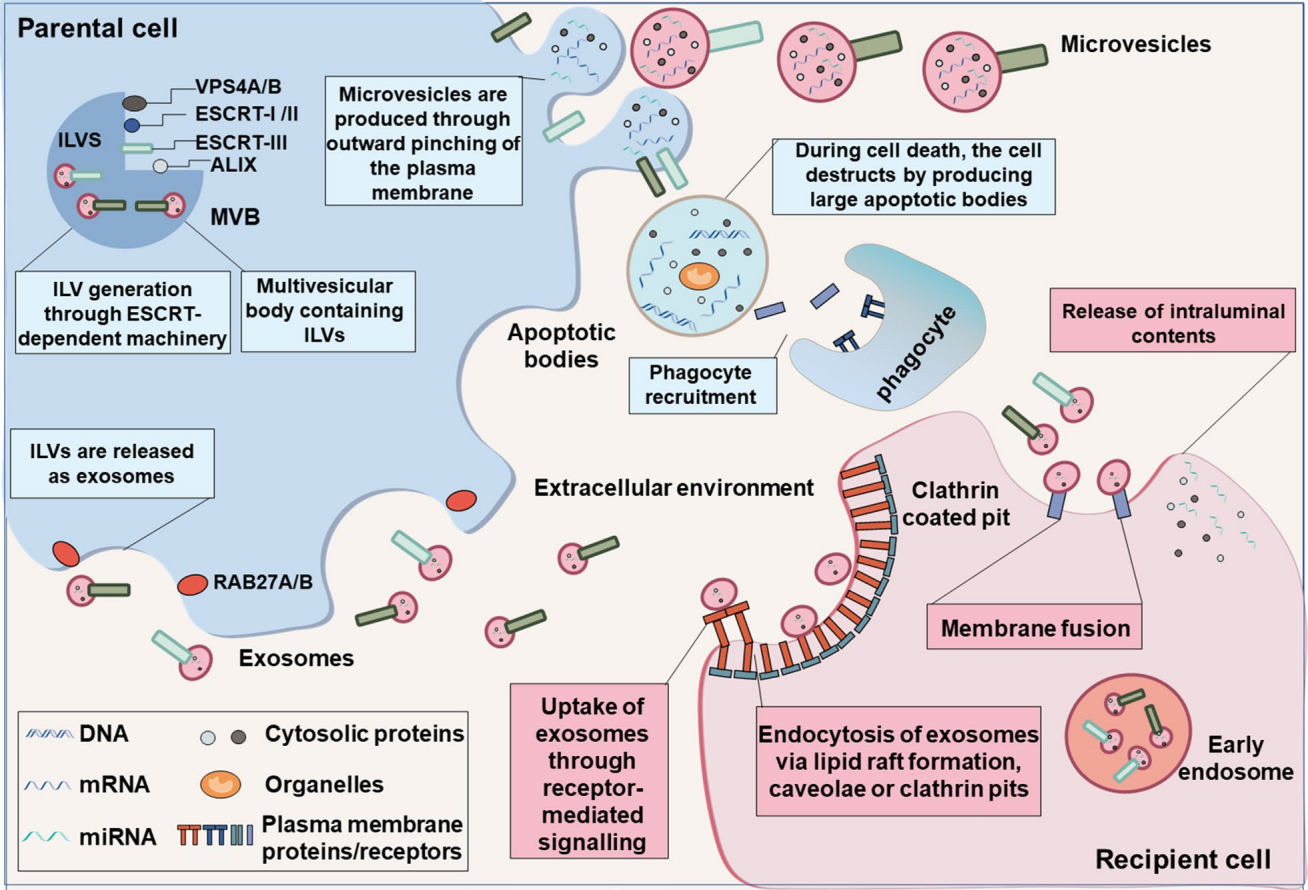
Classes, characteristics and uptake mechanisms of EVs. Various types of EVs are generated via distinct biological pathways. Exosomes are formed through the endosomal pathway, involving the ESCRT machinery, facilitating the invagination of intraluminal vesicles (ILVs) within multivesicular bodies (MVBs), followed by fusion with the plasma membrane and subsequent release into the extracellular environment. Microvesicles are formed by budding from the plasma membrane, encapsulating nearby proteins and nucleic acids. Apoptotic vesicles are produced during programmed cell death, wherein cells disassemble, randomly enclosing extracellular components within large apoptotic bodies. The apoptotic bodies are cleared by phagocytes. Microvesicles and exosomes undergo internalization via diverse mechanisms, including lipid raft formation, caveolae or clathrin pit endocytosis, and receptor-mediated signaling. Internalized EVs release their luminal contents into recipient cells, facilitating intercellular communication under physiological conditions or contributing to disease pathogenesis

EVs are primarily categorized into three major groups based on their biogenesis: exosomes, microvesicles, and apoptotic bodies [15, 16] (Fig. 1). Small exosomes typically range from 50 to 150 nm in diameter and contain heterogeneous populations of “classical” or “non-classical” exosomes. Exosomes originating from the classic endosomal route involve double invagination of the plasma membrane and formation of intracellular multivesicular bodies (MVBs), and transport by endosomal sorting complex required for transport (ESCRT) [17, 18]. RAS-related proteins RAB27A and RAB27B are essential for the secretion of exosomes from the plasma membrane [19], with small EVs concurrently abundant with tetraspanins CD63, CD9, and CD81 [20]. Recent in-depth studies have shown that mitochondria can produce small EVs with diameters ranging 70–150 nm [21], derived from both the outer and the inner mitochondrial membranes [22]. These vesicles, termed mitochondrial-derived vesicles (MDVs), facilitate communication between mitochondria and other organelles by transporting mitochondrial components to vesicles within other organelles [23]. Microvesicles are generated through cell membrane outgrowth, extending in size from 150 nm to over 1000 nm, typically averaging between 250 and 400 nm [2], categorized as large EVs [24]. Microvesicles are commonly distinguished by their lipid components and plasma membrane receptors [25, 26]. Microvesicle cargoes comprise biomolecules transported to the plasma membrane, frequently containing fragmented ribosomal RNA (rRNA) and mRNA [25, 27]. Apoptotic bodies, the largest EV category, range from 1 to 5 μm in size and are formed through apoptotic cell membrane blebbing and protrusion during cell death [28]. Typically, macrophages clear apoptotic bodies by identifying plasma membrane markers and subsequently initiating phagocytosis [29]. Compared to microvesicles and exosomes, apoptotic bodies exhibit a relatively complex structure, containing chromatin, significant amounts of 18S and 28S rRNAs, and chemokines that attract phagocytes for phagocytosis [30–32]. Recent studies have unveiled various mechanisms for EV formation, protein and RNA cargo sorting and generation of EVs with precise biochemical compositions [20, 33, 34]. However, many academic studies still interchangeably utilize the terms “exosomes” and “vesicles” due to incomplete comprehension of extracellular vesicle biogenesis and inconsistent purification protocols [35]. There is a dearth of comprehensive characterization regarding microvesicles. Hence, we employ “extracellular vesicles” as an umbrella term for both vesicle types.

## Multifaceted roles of EVs in intercellular communication and physiological processes

EVs directly activate cell surface receptors through proteins and biologically active lipid ligands, translocating their cargoes to recipient cells [36–38]. In this way, EVs are involved in the maintenance of normal physiological functions such as tissue repair [39] and immune surveillance [40, 41]. Thus, they can be regarded as signaling bodies governing multifunctional complexes for basic cellular and biological processes. Within the brain, intercellular neuronal communication is mediated through release of EVs, influencing various neurobiological functions including synaptic plasticity [42, 43]. Enhanced glutamatergic activity in neurons in the cortex is facilitated by the increased release of EVs containing neurotransmitter receptors [44]. In the regulation of immune responses, depending on the state of a particular immune cell, EVs may trigger an adaptive immune response or suppress inflammation in a tolerant manner [45, 46]. The diverse range of cellular and biological functionalities of EVs suggest their intrinsic therapeutic potentials, which will be discussed in following sections.

### Pathological roles of EVs in nervous system propagation and regulation

EVs play a critical role in regulating biological processes and, in certain instances, are equally important in disease pathogenesis. Numerous studies have shown the involvement of EVs in the transmission of pathogens, including HIV-1 entry into cells via lateral transfer of CC chemokine receptor 5 [47, 48], transferring Epstein–Barr virus (EBV) microRNAs (miRNAs) to inhibit the expression of EBV target genes in non-infected cells [49, 50], and selectively delivering prion protein with certain modifications and glycoforms to nerve cells [51, 52]. Furthermore, EVs may facilitate local propagation of disease-causing molecules in NDDs, influencing local synaptic plasticity and distant neuronal networks [53]. This phenomenon is clearly illustrated in Alzheimer’s disease (AD), where pathogenic amyloid-β (Aβ) peptide has been identified to be released in association with EVs, resulting in the accumulation of pathogenic Aβ in other brain regions [54, 55]. Similarly, α-synuclein has been detected in EVs, facilitating its transmission from enteric neurons to the brainstem and higher cortical centres [56].

## Brain cells are the source of EVs

Although detection of EV production in vivo at the tissue level is awaiting technological advancements, the presence of circulating EVs in vivo suggests their production by specific tissues and cells [57]. Various cell types in the brain, including neurons, astrocytes, microglia, oligodendrocytes, pericytes, and endothelial cells, have been shown to release EVs into the peripheral circulation [58]. The present review focuses on neuron-derived EVs (NDEs), astrocyte-derived EVs (ADEs), microglia-derived EVs (MDEs), oligodendrocyte-derived EVs (ODEs), pericyte-derived EVs, and endothelial cell-derived EVs (EDEs) **(**Table 1**)**. Surface markers for different cells are currently used to isolate peripherally circulating EVs secreted by brain-specific cells, from plasma or serum. Notably, antibodies for L1 cell adhesion molecule (L1CAM) [59–61], NCAM (neural cell adhesion molecule) [62, 63], GluR2/3 (α-amino-3-hydroxy-5-methyl-4-isoxazolepropionic acid subtype ionotropic glutamate receptor subunit 2/3) [64], synaptosome-associated protein 25 (SNAP25) [65, 66], and ATPase Na1/K1 transport subunit A3 [67] have been employed for non-invasive detection and isolation of NDEs. Plasma NDEs have been efficiently isolated from independent cohorts using markers L1CAM and SNAP-25, and proven to be highly consistent by miRNA cargo analysis [68]. However, concerns have been raised regarding the specificity of L1CAM [69]. In response, the ISTAART-BBB-PIA-EWG proposed an alternative solution for enriching NEVs from blood based on immunoprecipitation (IP). Specifically, they identified SYT1, SYP, NRXN2, and GRIA4 as potential novel neuronal markers for IP-based capture methods for NEV enrichment. They further uncovered candidate proteins of CNS origin that lack an extracellular domain, which are likely cargo proteins of EVs that could be used during secondary validation [70]. In summary, the identified candidate proteins exhibit diverse biological functions and may be related to neurodegeneration, but further research is needed.

**Table 1:** BDE isolation markers and cargoes analyzed for different neurodegenerative diseases

Disease classification	BDEs	Markers used for BDE isolation for cargo analysis	Cargo analysis			References
			Protein		miRNA	
Alzheimer’s disease	NDEs	L1CAM (CD171), NCAM, ATP1A3, GAP43, NGLN3	Tau, p-tau-T181, p-tau-S396, Aβ1–42, NRGN, REST, GAP43, cathepsin D, LAMP1, synaptotagmin1, pSer312-IRS-1, pYIRS-1, SNAP-25, PSD-95, GluR2, Syntaxin-1, AMPA4, NLGN1, hemoglobin, IL-6, MMP-9		miR-132, miR-212, miR-23a-3p, miR-223-3p, miR-190a-5p, miR-9-5p, miR-29a-5p, miR-106b-5p, miR-125b-5p, miR-132-5p, miR-135b-5p, miR-210-3p, miR-373, miR-204, Let-7e, miR-185	[8, 67, 97–117]
	ADEs	GLAST (ACSA-1), LRP-1	BACE1, γ-secretase, sAPPα, sAPPβ, p-tau-T181, p-tau-S396, Aβ1–42, LRP1, Cb3, C5b-c9		miR-29a-5p, miR-107, miR-125b-5p, miR-132-5p, miR-210-3p	[8, 99, 105, 107, 118–120]
	MDEs	CD11b, TMEM119	FTH1, TREM2, tau		miR-28-5p, miR-651-5p, and miR-188-5p, miR-29a-5p, miR-106b-5p, miR-132-5p, miR-210-3p, miR-34a	[8, 81, 121]
	ODEs	PDGFRα, MOG, CNPase			miR-29a-5p, miR-107, miR-125b-5p, a miR-135b-5p	[8]
	PDEs	PDGFRβ			miR-9-5p, miR-125b-5p, miR-132-5p, miR-210-3p	[8]
	EDEs	CD31, CD146	LAT-1, Aβ40, Aβ42, PrPc, p-tau-T181		miR-29a-5p, miR-125b-5p, miR-135b-5p, miR-210-3p	[90, 122]
Parkinson’s disease	NDEs	L1CAM (CD171), NCAM	α-Synuclein, tau aggregates, syntaxina- 1A, VAMP-2, SNAP-25, DJ-1, Rab8b, Rab31, PARK7		miR-19a-3p, miR-155	[123–128]
	ADEs	GLAST (ACSA-1),			miR-200a-3p	[129]
	MDEs	CD11b, TMEM119	α-Synuclein, MHCII, TNF-α			[56, 130, 131]
	ODEs	MOG	α-Synuclein			[83]
Amyotrophic lateral sclerosis	NDEs	SNAP25, L1CAM	Mutant SOD1, DPRS, TDP-43		miR1207-5p, miR-4739, miR-4505, miR24-3p, miR-1268a, miR-4507, miR-3176, miR150-3p	[132–134]
	ADEs	GLAST (ACSA-1), LRP-1	Mutant SOD1, IL6,		miR-494-3p, miR-200a-3p, miR-21	[135–139]
	MDEs	CD11b, TMEM119	Mutant SOD1, HMGB1		miR-155, miR-146a	[140, 141]
Huntington’s disease	NDEs	L1CAM (CD171)	mHTT			[142]
	ADEs	GLAST (ACSA-1), LRP-1	CRYAB			[143]
Multiple sclerosis	NDEs	L1CAM (CD171), NCAM	MAP1b			[144]
	ODEs	MOG	PLP, MBP, MOG, CNP		Let-7i, miR-19b, miR-25, miR-92a	[145, 146]
	EDEs	CD31, CD146	b2-microglobulin, MHCII, CD40, ICOSL			[147]

*ATP1A3* ATPase Na1/K1 transport subunit A3, *AMPA4* AMPA-type glutamate receptor subunit 4, *CNPase* 2,3-cyclic nucleotide-3-phosphodiesterase, *cathepsin D* Lysosomal aspartyl protease, *Cb3* Cytochrome b3, *C5b-c9* C5b-9 complex, *CD11b* Cluster of differentiation 11b, *CD31* Cluster of differentiation 31, *CD146* Cluster of differentiation 146, *CRYAB* Alpha-B crystallin, *CNP* 2',3'-Cyclic nucleotide 3'-phosphodiesterase, *CD40* Cluster of differentiation 40, *DJ-1* Park7 protein, *DPRS* Dipeptide repeat proteins, *FTH1* Ferritin heavy chain 1, *GAP43* Growth-associated protein 43, *GluR2* Glutamate ionotropic receptor AMPA type subunit 2, *HMGB1* High mobility group box 1, *ICOSL* Inducible T-cell co-stimulator ligand, *MHCII* Major histocompatibility cComplex class II, *LAMP1* Lysosomal-associated membrane protein 1, *LRP-1* Low-density lipoprotein receptor-related protein 1, *LAT-1* L-type amino acid transporter 1, *mHTT* Mutant huntingtin protein, *MBP* Myelin basic protein, *MMP-9* Matrix metallopeptidase 9, *MAP1b* Microtubule-associated protein 1B, *MOG* Myelin oligodendrocyte glycoprotein, *NCAM* Neural cell adhesion molecule, *NGLN3* Neuroligin-3, *NRGN* Neurogranin, *NLGN1* Neuroliglin 1, *PDEs* Pericyte-derived EVs, *PDGFR* CD140a/platelet-derived growth factor receptor, *pSer312-IRS-1* Phosphoserine 312 insulin receptor substrate 1, *pYIRS-1* Phosphotyrosine insulin receptor substrate 1, *PSD-95* Postsynaptic density protein 95, *PDGFRα* Platelet-derived growth factor receptor alpha, *PDGFRβ* Platelet-derived growth factor receptor beta, *PrPc* Prion protein, cellular, *PARK7* Parkinson protein 7, *PLP* Proteolipid protein, *REST* RE1-silencing transcription factor, *Rab8b* Ras-related protein Rab-8B, *Rab31* Ras-related protein Rab-31, *SNAP25* Synaptosome-associated protein 25, *SOD1* Superoxide dismutase 1, *sAPPα* Soluble amyloid precursor protein alpha, *sAPPβ* Soluble amyloid precursor protein Bbeta, *TNF-α* Tumor necrosis factor-alpha, *TDP-43* TAR DNA-binding protein-43, *TREM2* Triggering receptor expressed on myeloid cells 2, *VAMP-2* Vesicle-associated membrane protein 2 Gamma-secretase

Non-neuronal cell-derived EVs are also receiving much attention and can provide insights into the pathophysiological features of different neuropsychiatric diseases. Astrocytes are crucial for neuroprotection, neuroregeneration, repair, and maintenance of normal neuronal function. Notably, ADEs are involved in various neuropathological conditions [71–74]. Biotinylated antibodies for glutamine aspartate transporter (GLAST) are predominantly used for non-invasive detection of isolated ADEs [75, 76]. Similarly, microglia play a fundamental role in maintaining CNS integrity and functionality [77, 78]. Recently, Kumar et al. have demonstrated the use of microglia-specific transmembrane protein 119 (TMEM119) antibody conjugated to streptavidin-coated magnetic beads for the isolation of MDEs from plasma [7]. Recent studies have enriched NDEs, ADEs, and MDEs using biotinylated L1CAM, GLAST, and TMEM119 antibodies (affixed to streptavidin-coated magnetic beads), respectively, and separation of the magnetic bead-antibody-EV complexes by flow cytometry [79, 80]. Concurrently, CD11b can be utilized for the isolation of MDEs from cryopreserved brain tissues [81]. Myelin oligodendrocyte glycoprotein (MOG), CD140a/platelet-derived growth factor receptor α (PDGFRα), and CNPase (2,3-cyclic nucleotide-3-phosphodiesterase) have demonstrated potential use for isolating ODEs [8, 82, 83]. Several challenges remain regarding the isolation of brain-derived, cell-specific EVs from peripheral fluids. Recent studies have shown that TMEM119^+^ MEVs in the blood are diluted by TMEM119^+^ EVs from peripheral monocytes and macrophages [84], raising concerns on the use of TMEM119 for isolating MDEs. Similarly, another study demonstrated that L1CAM present in human plasma is not associated with EVs, but exists in the soluble fraction [69], further questioning whether non-neural EV contamination occurs when using L1CAM antibodies alone for isolation. Additionally, the concentrations of neurogenic EVs in peripheral blood are typically low. The isolation of cell lineage-specific brain cell-derived extracellular vesicles (BDEs) is not only limited by the specificity of available markers, but also demands advanced extraction and purification techniques. Development of more precise markers is urgently needed. In many studies, EV preparations may contain trace amounts of non-target EVs, as the surface markers are not entirely exclusive. Most studies rely on enzyme-linked immunosorbent assay (ELISA) to quantify marker levels in isolated exosome preparations. It remains uncertain whether variations of levels of cell surface proteins used for isolation, particularly those driven by underlying biological processes, could influence measurement of concentrations. The effects of disease states and stages on EV production are still poorly understood.

Similar to glial cells, brain pericytes and endothelial cells could actively contribute to the pathogenesis of diseases, forming a cohesive unit with neurons and glial cells [85–88]. Recent studies have emphasized the potential use of EDEs in understanding cerebrovascular diseases and cognitive functions [89, 90]. For instance, Abner et al. reported the separation of EDEs from plasma using surface markers CD31 and CD146 to investigate the impact of cerebellar vascular diseases on AD progression [90]. In NDDs, mitochondrial dysfunction leads to increased release of MDVs. These vesicles are typically enriched with mitochondrial DNA (mtDNA) and other damage-associated molecular patterns, which can lead to neuroinflammation [91, 92]. The identification of MDVs often involves confirmation of their mitochondrial origin by detecting mtDNA within EVs [93]. This is commonly achieved through the use of antibodies for mitochondrial markers such as TOMM20 (translocase of the outer mitochondrial membrane complex subunit 20), ATP5A (ATP synthase subunit 5A), MTCOI (mitochondrial cytochrome c oxidase subunit I), NDUFB8 (NADH:ubiquinone oxidoreductase subunit B8) and core subunit S3 (NDUFS3 (NADH:ubiquinone oxidoreductase core subunit S3), SDHA (succinate dehydrogenase complex flavoprotein subunit A), SDHB (succinate dehydrogenase complex iron sulfur subunit B), and UQCRC2 (ubiquinone-cytochrome c reductase core protein 2) [94–96]. Investigations into the cargoes of BDEs have primarily focused on proteins and miRNAs/mRNA, with limited exploration of lipid content. Table 1 summarizes techniques for characterizing BDEs and their cargo proteins and miRNAs under various neurological conditions. In the following sections, we will delve into research exploring BDEs within diverse disease contexts.

## Biological mechanisms of BDEs in neurodegenerative diseases

### AD

AD is a NDD influenced by both genetic and environmental factors [148]. Neuroinflammation, brain-gut axis dysregulation, and metabolic abnormalities are also associated with AD [149]. Currently, the diagnostic standard for AD necessitates invasive cerebrospinal fluid (CSF) analysis and costly neuroimaging, typically at a mid-to-late disease stage. Less invasive and cost-effective early diagnostic indicators for AD are needed. Studies suggest that as AD progresses, the production and function of EVs undergo alterations. Blocking EV release leads to substantial relief from AD phenotype [150]. This underscores the significant role of EVs in the pathogenesis of AD [151].

Brain cell type-specific EVs in peripheral biological fluids offer a window for understanding the molecular alterations in the brain during AD. Alterations of AD-related miRNA levels are observed in EVs extracted from SH-SY5Y cells expressing the Swedish mutation of APP695 (SHSwe) and mouse neuroblastoma N2a cells expressing human amyloid precursor protein (APP) [152, 153]. NDEs isolated from the blood of AD patients have demonstrated notable neurotoxicity towards rat primary cortical neurons in contrast to NDEs from healthy donor plasma [107]. The neuronal impairment induced by NDEs might be mediated by the transmission of AD-associated molecules [154, 155]. In an in vitro AD model, EVs from SHSwe cells are internalized by microglia, delivering miR-155, miR-146a, miR-124, miR21, and miR-125b, triggering neuroinflammation [152]. However, recent findings indicate that N2a cells release EVs rich in miR-185 in vitro, which inhibit APP expression in recipient N2a cells, suggesting that NDEs possess potential anti-Aβ-deposition properties [153]. These findings imply that NDEs exhibit dynamic alterations during the progression of AD.

Astrocytes are closely related to p-tau and Aβ, and ADEs have emerged as one of the significant factors in NDD pathogenesis [120]. In a co-culture environment, astrocytes with excessive Aβ accumulation produce elevated p-tau levels, leading to the release of ADEs that aggregate Aβ-induced neurotoxicity, ultimately resulting in neuronal loss in AD cells and animal models [156]. In addition, ELISA revealed markedly elevated levels of beta-secretase 1 (BACE1) and complement proteins in ADEs isolated from plasma and CSF [118, 120]. BACE1 is an Aβ-secretase enzyme that contributes to APP cleavage to produce Aβ peptides, while C3b and C5b-C9 complexes may exacerbate neuronal damage through microglia-mediated neurotoxic reactions [157, 158]. These findings propose that, in addition to p-tau effects, ADEs could influence Aβ accumulation and induce neurotoxicity by altering Aβ-processing enzymes and pro-inflammatory factors. Interestingly, ultrasound-mediated ADE release alleviates Aβ-induced neurotoxicity [159]. The potential beneficial impact of ADEs on AD progression in vivo warrants further investigation.

Microglia play either neurotoxic or neuroprotective roles within different microenvironments. Pathological microglia release EVs containing pathogenic molecules to facilitate formation of disease-associated microenvironments in NDDs [160]. MDEs play a crucial role in AD by encapsulating key AD pathogenic factors like Aβ and tau, enabling their transmission between cells [161, 162]. Recent studies have demonstrated that Aβ can be stored in the lumen or on the surface of MEVs [163]. In 2014, Joshi et al. were the first to provide evidence of neurotoxicity of Aβ-carrying EVs released by microglia of AD patients [164]. Other studies have indicated that EVs carrying prion proteins on the surface play an important role in Aβ-mediated neurodegeneration. Prion proteins on the surface of neuronal cells act as Aβ receptors and activate neurotoxic signaling [165]. Furthermore, lipid components of MDEs promote formation of soluble Aβ species from extracellular insoluble aggregates, which significantly enhance Aβ neurotoxicity [164]. In addition, engulfed tau in microglia is loaded into MDEs, which further transport tau to neurons, leading to abnormal tau accumulation within neurons [166, 167]. Sequencing analysis revealed that MDEs from AD patient brains are notably enriched in inflammation- and cellular senescence-related miRNAs that are implicated in the pathogenesis of AD [81]. Notably, Li et al. demonstrated that MDEs could enhance nerve cell viability and mitigate mitochondrial dysfunction in vitro, along with reducing in vivo Aβ deposition, highlighting the beneficial potential of MDEs for AD [168]. Additionally, Huang et al. reported that TREM2 (triggering receptor expressed on myeloid cells 2) on the surface of MDEs binds to Aβ, altering the Aβ-associated inflammatory environment and enhancing Aβ phagocytosis by microglia. The MDE-mediated microglia-Aβ interaction expedites Aβ clearance [169]. Moreover, MEVs with surface Aβ impair synaptic plasticity both in vitro and in an entorhinal cortex slice model of mice with intact neuronal circuitry. When injected into mice, these EVs induce synaptic dysfunction in the entorhinal-hippocampal circuit through an annexin-V-sensitive mechanism [162]. By characterizing systemic EVs, Villar-Vesga et al. have identified increases of endothelial- and leukocyte-derived EVs containing mitochondrial markers in patients with sporadic AD [170]. Additionally, Kim et al., using RNA-Seq analysis, reported elevated levels of mitochondrial RNAs (mtRNAs), including MTND1-6 mRNA and other protein-coding and non-coding mtRNAs, in plasma EVs from patients with mild cognitive impairment and AD, compared to healthy controls [171]. In summary, EVs are recognized as a pivotal element within the AD pathological microenvironment, where disruptions of EV release and cargo sorting dramatically influence AD pathogenesis and progression.

## PD

PD is a neurodegenerative disorder characterized by degeneration of dopaminergic neurons, resulting in the progressive loss of autonomic control, predominantly in older adults [172, 173]. Polymorphic α-synuclein carried by EVs has emerged as pivotal factors mediating dopaminergic neuron degeneration. Studies in neuronal models of PD have demonstrated the presence of α-synuclein in NDEs [123, 124], which facilitate the spread of α-synuclein between cells, leading to α-synuclein oligomerization in normal neurons and impairing the autophagic function of microglia [126], thereby exacerbating the disease process. SUMOylation has been identified to mediate loading of α-synuclein into EVs through membrane interactions [174]. A study of 664 serum samples from patients with NDDs found a significant increase of α-synuclein in NDEs from PD patients compared to controls or patients with other NDDs. Importantly, α-synuclein in NDEs is elevated in early disease stages and continues to rise as the condition progresses [175]. Moreover, PD-associated miRNAs have been identified in NDEs from PD patients and in EVs in cellular models of PD. Specifically, miR-19a-3p in NDEs represses microglial autophagic function by targeting the PTEN/AKT/mTOR signaling pathway [126]. Notably, the expression of lnc-MKRN2-42:1 in plasma EVs of PD patients correlates positively with scores on the Revised Unified PD Rating Scale III [176]. Similarly, lnc-POU3F3 levels in plasma NDEs positively correlate with motor dysfunction or non-motor symptoms in individuals with PD [177].

In PD, astrocytes and microglia participate in the clearance of pathological α-synuclein through endocytosis, where excessive accumulation of α-synuclein can trigger inflammatory responses in astrocytes and microglia, leading to the release of ADEs and overproduction of MDEs [131]. Additionally, EVs released by astrocytes carrying PD-associated *LRRK2* G2019S mutation, although unchanged in quantity, are unable to provide comprehensive neurotrophic support after being internalized by dopaminergic neurons, implying changes of enriched cargo contents within ADEs that may contribute to PD progression [178]. Notably, α-synuclein oligomers have been detected in MDEs isolated from the CSF of PD patients and EVs obtained from preformed fibril (PFF)-treated microglia. Injection of exosomes isolated from microglia treated with PFFs into the striatum of mice induces dopaminergic neuron degeneration and associated behavioral alterations [56]. Moreover, EVs generated under inflammatory conditions simulating PD through microglial exposure to α-syn/interferon-γ (IFN-γ)/lipopolysaccharide (LPS) exhibit heightened levels of major histocompatibility complex (MHC) class II molecules and tumor necrosis factor α, initiating dopaminergic neurodegeneration, suggesting a complex mechanism for MDE-mediated PD pathogenesis and progression [130, 131]. Oligodendrocytes play a vital role in facilitating rapid nerve conduction within the CNS by producing myelin. EVs released by myelinating oligodendrocytes are internalized by neurons to sustain axonal transport [179]. Brain-derived ODEs isolated from the plasma or serum of PD and MSA individuals by immunoprecipitation using anti-MOG antibody showed elevated levels of α-synuclein, suggesting a potential role of brain-derived ODEs in facilitating α-synuclein spread throughout the CNS [83]. MDVs also play an important role in PD. Wang et al. showed that *VPS35* mutations in sporadic PD patients lead to enhanced VPS35–dynamin-like protein 1 (DLP1) interactions, which in turn improve turnover of the mitochondrial DLP1 complex via MDV-dependent transport of the DLP1 to lysosomes for degradation, leading to excessive fission and consequently mitochondrial dysfunction [180].

## ALS

ALS is characterized by the gradual degeneration and death of motor neurons, leading to rapid atrophy of the medulla oblongata, limbs, and respiratory muscles [181]. Currently identified potential mechanisms of ALS consist of aberrant RNA processing, superoxide dismutase 1 (SOD1) toxicity, cytoskeletal disorders, mitochondrial dysfunction, viral infections, among others [182, 183]. EVs have been discovered as a crucial component of the pathological microenvironment, contributing significantly to the pathogenesis of ALS [120].

A recent study showed that SNAP25-positive EVs extracted from brains and spinal cords of ALS mice contain abundant misfolded and neurotoxic SOD1 [132]. The presence of mutated SOD1 within NDEs initiates a pro-inflammatory response in microglia and disrupts microglial autophagy upon internalization [184]. Moreover, EVs released from spinal motor neurons derived from pluripotent stem cells from* C9orf72*-ALS patients contain dipeptide repeat proteins and TAR DNA-binding protein-43 (TDP-43) [133]. Concurrently, altered miRNA composition of NDEs in plasma of ALS patients has been observed [185]. NDEs isolated from ALS patients contain significantly elevated levels of miR-24-3p compared to controls, potentially affecting neuroplasticity and exacerbating neural injury by modulating BOK and CHD5 levels [186, 187]. Conversely, notable downregulation of miR-150-3p was detected, which may confer neuroprotection by targeting CASP2 [188]. These results highlight the association of NDEs with ALS progression. Additionally, proteins involved in the regulation of synaptic and axonal membranes are diminished in BDEs collected from ALS patients [189]. Nevertheless, further studies are warranted to confirm the effects of NDEs.

In ALS models, SOD1 was observed to be encapsulated in both ADEs and MDEs. These mutant SOD1 proteins are transferred from glial cells to neuronal cells via EVs, resulting in their accumulation, which triggers neurotoxicity and nerve damage [135, 136, 190]. Additionally, EVs secreted by astrocytes derived from pluripotent stem cells of ALS patients carrying *C9orf72* mutations contain reduced level of miR-494-3p [138]. This downregulation negatively impacts the expression of *SEMA3A*, a gene related with axon maintenance, contributing to neuronal degeneration in ALS [191]. Notably, elevated interleukin-6 (IL-6) levels were detected in ADEs isolated from the plasma of sporadic ALS patients, highlighting the role of pro-inflammatory factors carried by ADEs in initiating and aggravating neuroinflammation in ALS [137]. Furthermore, the levels of high mobility group box 1 protein (HMGB1), miR-155, and miR-146a are notably increased in EVs originating from microglia overexpressing mutant *SOD1* [140]. The HMGB1/RAGE (receptor for advanced glycation end products) axis has been implicated in neuroinflammation by disrupting mitophagy flux in microglia [192], while miR-155 acts as a pro-inflammatory miRNA regulating microglial activation [193]. Consequently, the presence of these pro-inflammatory molecules in MDEs can lead to neuroinflammation, exacerbating the ALS phenotype.

## HD

HD is an autosomal-dominant inherited neurodegenerative disorder caused by abnormal expansion of a cytosine-adenine-guanine (CAG) trinucleotide repeat within the huntingtin (*HTT*) gene on chromosome 4p [194]. Emerging research has indicated that the EV-mediated transport of neurotoxic polyglutamine (polyQ) proteins and expanded repeat RNAs is a key mechanism contributing to the progression of HD [195].

Recent studies have revealed *HTT* overexpression in neurons in the brains of HD patients [196]. In a previous study, EVs released by human 293T cells expressing an expanded CAG repeat were found to contain both CAG-repeat RNAs and mutant HTT peptides. These EVs transferred CAG-repeat RNAs to a mouse striatal cell line [197]. In addition, 111/111Q striatal cells can also incorporate CAG-repeat RNAs into EVs [198]. These findings imply the involvement of NDEs in the propagation of mHTT in the brain, although conclusive evidence is still required. Additionally, a more recent study has shown the potential use of NDEs for HTT suppression by delivering HTT-targeting miRNAs [198]. Nonetheless, further investigation is essential to elucidate the pathological role of NDEs in HD.

To date, limited research has focused on the role of ADEs in HD pathogenesis. Deep sequencing of highly expressed genes in ADEs has revealed their roles in promoting HD pathology [199]. mHtt in astrocytes decrease the expression of αB-crystallin (CRYAB) which facilitates AEV secretion, by disrupting the binding of Sp1 to the CRYAB enhancer, consequently impeding ADE secretion. Additionally, mHtt also inhibits CRYAB sorting into ADEs, triggering glial activation and neuroinflammation [143], ultimately contributing to neurodegeneration in HD.

## Multiple sclerosis (MS)

MS is a chronic autoimmune disorder affecting the CNS, featured with inflammation, cellular infiltration, myelin degeneration, oligodendrocyte apoptosis, and axonal damage [200]. Recent studies have highlighted the role of EVs in preserving synaptic plasticity [201, 202]. Specifically, EVs released by depolarizing neurons exhibit elevated levels of MAP1b, a key factor in synaptic plasticity and myelin production [144]. Antonucci et al. revealed that MDEs stimulate synaptic activity via enhanced sphingolipid metabolism in neurons [203]. Moreover, the presence of synaptophysin-1 in ADEs suggests their involvement in neural survival during development [204]. MDEs also modulate inhibitory neural transmission by activating CB1R on GABAergic neurons [205]. Additionally, ODEs are abundant in myelin-forming proteins [145]. These vesicles impede the differentiation of oligodendrocyte progenitor cells (OPCs) and hinder myelin synthesis by activating ROCK (Rho-associated coiled-coil protein kinase) and inducing actomyosin contraction during CNS development [206]. Subsequently, researchers identified 16 differentially expressed miRNAs in EVs derived from MS patients, with a focus on 4 miRNAs—let-7i, miR-19b, miR-25, and miR-92a, suggesting their potential use as prognostic biomarkers and as indicators to monitor patient response to therapeutic interventions [207].

## BDEs as potential diagnostic markers for NDDs

EVs are released by various cell types and are present in biological fluids, making them highly suitable for liquid biopsy applications. BDEs can be isolated from CSF. They can also cross the BBB [208, 209], allowing for detection in peripheral body fluids. In recent years, numerous methods for EV isolation have been developed, ranging from filtration and ultracentrifugation to microfluidic arrays [210, 211]. Nonetheless, only a few methods can extract adequate quantities of EVs from patient samples. A significant challenge remains in verifying whether the isolated EVs originate from the intended cell type, as some markers, like L1CAM, are not exclusively expressed in neurons. Currently, there are nearly 30 clinical trials registered on ClinicalTrials.gov investigating the potential of EVs as biomarkers for various diseases, including obesity, cancer, cardiovascular diseases, and NDDs.

Additionally, combining multiple markers, such as CSF p-tau and Aβ, may enhance the diagnostic sensitivity and specificity [116]. Combined with protein analysis, profiling EV miRNAs may offer more precise insights into the pathogenesis and progression of diseases [212, 213]. In recent years, numerous clinical trials have investigated the potential of EVs as novel biomarkers for AD diagnosis or monitoring therapeutic response. In 2017, researchers from the University Hospital of Lille analyzed tau levels in EVs extracted from the CSF of AD patients (ClinicalTrials.gov Identifier: NCT03381482). In 2019, a group from Oxford University tested free protein biomarkers and EV numbers to assess the effects of JNJ-40346527 on microglial activity in the brain. In the same year, researchers from the National Institute on Aging in Baltimore separated total EVs and NDEs from plasma to evaluate whether the anti-diabetic drug empagliflozin increases ketone body levels and promotes neuronal health, potentially delaying cognitive decline. These ongoing studies propose innovative approaches to identifying non-invasive diagnostic and prognostic biomarkers of neurodegeneration.

A recent study analyzing miRNAs in plasma EVs from PD patients revealed that the target genes of the differentially expressed miRNAs are enriched in Gene Ontology (GO) and Kyoto Encyclopedia of Genes and Genomes (KEGG) pathways including dopaminergic synapse, regulation of neurogenesis, and neuron projection guidance, underscoring the significant role of these miRNAs in PD progression [214]. Despite the promise of EV miRNAs as biomarkers, standardized protocols for their clinical application are still lacking [215, 216]. Furthermore, the proportion of brain-derived miRNAs in the circulating pool of noncoding RNAs is very small, which may obscure significant differences when analyzing biological fluids from patients with neurological diseases. Therefore, standardizing protocols for BDE isolation is important for yielding useful biomarkers for NDDs through analyzing BDE-derived molecules. Currently, four clinical studies are investigating EVs as a source of PD-related biomarkers. Two of them (NCT03775447, Fox BioNet Project ECV-003, and NCT04603326, Fox BioNet Project ECV-004), sponsored by the Michael J. Fox Foundation for Parkinson’s Research, aim to optimize the isolation of EVs from human CSF to detect LRRK2 level and activity. The other two clinical trials, sponsored by the Don Carlo Gnocchi Onlus Foundation (NCT05452655 and NCT05320250), focus on testing a new set of serum biomarkers in NDEs for assessing rehabilitation outcomes in PD patients and validating molecules from saliva or salivary EVs as biomarkers for differentiating PD from atypical parkinsonism using Raman spectroscopy analysis.

## Potential therapeutic strategies to inhibit BDE formation and release

**Fig. 2:**
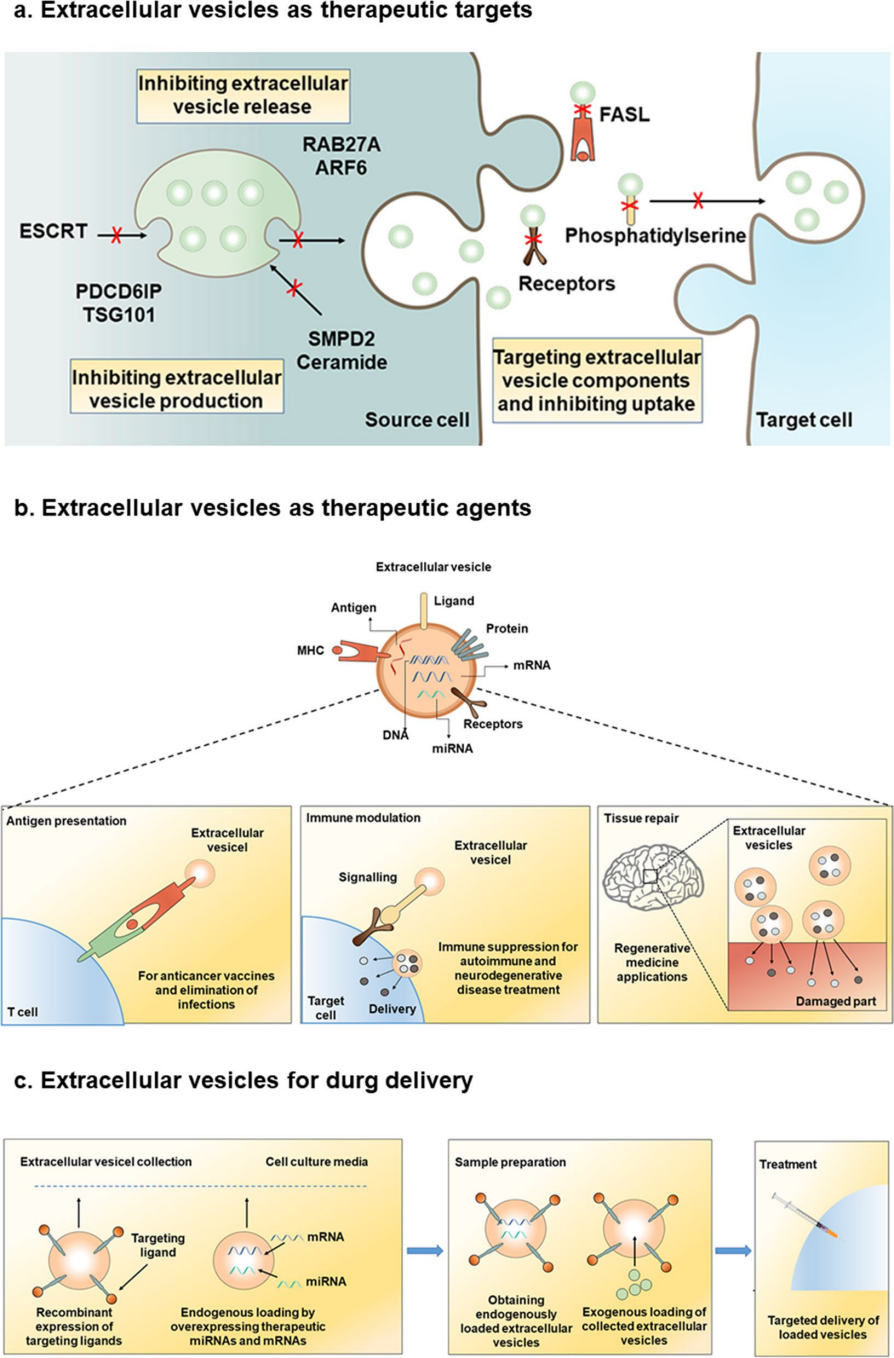
Therapeutic strategies targeting EVs and EV-based drug delivery. **a** The associations of EVs with various pathological processes suggest the potential of targeting EV production, release, or uptake for disease treatment. EV production can be controlled by inhibiting intracellular ceramide synthesis, disrupting synaptophysin-synaptoglycan interactions, or blocking tetraubiquitin. Inhibiting specific RAB GTPases or ADP ribosylation factor 6 (ARF6) can decrease EV release. In addition, interrupting the EV uptake signaling can be achieved by blocking specific receptors or lipids that bind to EVs. **b** EVs play a role in regulating normal physiological processes and thus hold promise as therapeutic agents in regenerative medicine and for tissue repair. EVs exert immune modulatory effects through antigen-specific or nonspecific mechanisms. Immunomodulatory effects of EVs may include antigen transfer and presentation: for example, in anticancer vaccines and in eliminating infections by activating specific CD8+ T cells. Since EVs can promote the release of pro-inflammatory cytokines from target macrophages, the overall immune activation properties may be important in cancer treatment and other immunotherapy applications. In models of cerebral ischemia–reperfusion injury and NDDs, lesion repair may rely on the transfer of EV-loaded growth factors, soluble proteins, bioactive lipids, and genetic materials. **c** Given that EVs inherently contain mRNAs and regulatory RNAs, they offer a means to deliver specific oligonucleotide drugs. Tissue-specific targeting by EVs can be achieved by expressing plasmid fusion constructs in cells during their production. Drug loading can be performed by endogenous or exogenous methods. Endogenous loading involves overexpression of miRNAs, short hairpin RNAs, or mRNAs in the parental cells, resulting in drug-loaded EVs upon collection. Exogenous loading methods include gathering drug-free EVs and loading them with desired cargoes, either through EVs transport peptides or via electroporation

Given that BDEs transport pathogenic proteins or neurotoxic molecules to recipient cells, inhibiting EV formation, release, or uptake may diminish the vesicle-mediated pathogenic burden. There are three main strategies for inhibiting EV release, including targeting neutral sphingomyelinase (nSMase 2) to inhibit EV release, targeting components of the ESCRT machinery, and blocking uptake of pathogenic EVs by recipient cells **(**Fig. 2a**)**. However, it is challenging to specifically target pathogenic EVs while not affecting healthy EVs to maximize the effectiveness of treatment.

The formation of EVs relies on various cellular components. Inhibiting ceramide synthesis, either through small-molecule inhibitors of neutral sphingomyelinase or by treatment with the blood pressure-lowering drug amiloride, has been shown to reduce EV production [217, 218]. Additionally, recent research highlights that endocytin interacts directly with the exosomal protein PDCD6IP (programmed cell death 6-interacting protein, also known as Alix). Disrupting this interaction by either RNA interference or small-molecule inhibitors can reduce EV release [219]. GW4869, which targets nSMase 2, has been used in various studies to impede EV release. Treatment of prion-infected cells with monensin leads to increased EV release, facilitating prion spread between cells, and this process can be reversed by GW4869 [220]. In primary microglia cultured from AD mouse models expressing human mutant tau, GW4869 or nSMase2-targeting small-interfering RNA (siRNA) inhibited tau propagation through MDEs [221]. In the 5 × FAD mouse model, GW4869 effectively reduced ceramide levels, resulting in decreased BDE levels and serum EV secretion. Although EV secretion may still occur via alternate pathways, the study underpins the potential of inhibiting nSMase2 to reduce amyloid and plaque burdens in AD brains in vivo [222]. A follow-up to this study involved nSMase2-deficient 5 × FAD mice showing a significant reduction in neuronal secretion of EVs in brain tissue compared to 5 × FAD mice [223]. By decreasing amyloid load and tau phosphorylation, cognition was improved in a 5 × FAD mouse model [223]. Further research is required to delve into the potential of targeting nSMase2 to decrease EV release. Meanwhile, it is crucial to investigate the potential off-target effects of GW4869. Notably, GW4869 possesses lipid-binding capabilities apart from its nSMase 2 inhibitory action [224]. Alterations of cellular expression of nSMase2 may present additional hurdles [225, 226]. Components of the ESCRT machinery can be targeted to inhibit the release of pathogenic EVs [227]. While the field is still developing, various components of the ESCRT machinery and its adjunct proteins may be of research interest. Knockdown of HRS (hepatocyte receptor tyrosine kinase substrate) in a cellular model of prion disease can impede the release of prion proteins, consequently reducing infectivity. By silencing *TSG101* (tumor susceptibility gene 101), a member of the ESCRT-I complex, researchers confirmed that transmission is facilitated through EV secretion [228]. Notably, inhibiting EV release in NDDs may result in the accumulation of neurotoxic proteins, accelerating pathological processes such as endosomal lysosomal dysfunction, a major contributory factor. For instance, silencing the RAS-related protein RAB27A has been associated with aggregation of TDP43, a characteristic feature of ALS [229].

Another avenue to inhibit EV-mediated disease transmission is blocking EV uptake pathway. Studies have shown that diannexin, which blocks surface phosphatidylserine, can reduce the uptake of EVs by cells [230, 231]. However, the applicability of this approach may be limited due to the roles of phosphatidylserine in other physiological functions, such as the clearance of apoptotic cells. In non-tumor contexts, targeting ICAM1 (intercellular adhesion molecule 1) on HIV-1 viruses encapsulated in EVs can hinder their spread to T cells by preventing binding to the specific integrin FLA1 [232]. Additionally, FASL-specific monoclonal antibodies have demonstrated potential in inhibiting tumor growth by blocking key signaling components of EVs, though this approach may lack specificity and could adversely affect the overall immune function [233]. Similarly, targeting the target moiety via RNAi to prevent its active incorporation into EVs may prove useful in ameliorating disease progression if sufficiently potent siRNA can be delivered to recipient cells in vivo [234]. However, the complex mechanisms of EV uptake are still incompletely characterized [2, 19]. Various mechanisms are being explored, such as endocytosis through clathrin-dependent and -independent pathways, plasma or endosomal membrane fusion, phagocytosis, and micropinocytosis. It is difficult to assess internalization and absorption due to the microscopic limitations. However, encouraging findings have been reported. For instance, the motility inhibitor dynosore inhibits transferrin internalization via the clathrin-mediated pathway. Notably, Nanbo et al. have observed that knockdown of caveolin-1 significantly reduced uptake and internalization of labeled CD63^+^ EVs from EBV-infected B cells, by EBV-negative epithelial cells [235].

The aforementioned methods primarily focus on inhibiting the transmission and uptake of pathogenic EVs. However, the crucial role of EVs released by healthy brain cells in maintaining homeostasis, supporting neurogenesis, promoting synaptic plasticity, facilitating myelination, and ensuring neuronal survival should not be overlooked [203, 236, 237]. Recent studies have demonstrated that neurons release EVs containing neurotransmitter receptors and activity-dependent molecules, which influence the excitability of neighboring cells and modulate synaptic plasticity [238, 239]. Additionally, under oxidative stress, neuron-oligodendrocyte interactions trigger the release of EVs from oligodendrocytes, which contain neuroprotective RNAs and proteins [240, 241]. EVs from healthy brain cells also contribute to the removal of unwanted proteins and macromolecules. In cases where the autophagy-lysosome pathway is impaired, EV secretion provides a compensatory pathway for clearance of TDP-43 aggregates [229]. Furthermore, neuronal EVs have been reported to facilitate the conversion of extracellular Aβ into non-toxic amyloid fibers, enhancing their internalization and degradation by microglia [242]. Therefore, it is essential to seek methods and compounds that selectively target pathogenic EVs while preserving the physiological protective functions of healthy EVs.

## Potentials and applications of EVs as drug delivery vehicles for neurological therapies

In comparison to liposomes, EVs injected exogenously in mice can infiltrate other cells and transmit functional cargoes with minimal immune response [16] (Fig. 2b). Additionally, EVs are well-tolerated, showing promising therapeutic potentials. Numerous in vivo studies have highlighted the therapeutic promise of EVs from various origins such as mesenchymal stem cells, neural stem cells, dendritic cells, macrophages, plasma-derived EVs, and biomimetic nanovesicles [243–246] (Fig. 2c).

### Mesenchymal stem cell (MSC)-derived EVs

MSCs can be obtained from diverse tissues, such as umbilical cord blood, bone marrow, dental pulp, and adipose tissue. Numerous clinical trials have been conducted to test the efficacy of MSC therapies and have demonstrated good tolerance and safety. It is hypothesized that the therapeutic effects of MSCs stem from secreted factors rather than the cells *per se* [247]. Consequently, there is significant interest in employing EVs derived from MSCs as promising therapeutic agents. Additionally, MSC-derived EVs offer numerous advantages, including minimal immunogenicity, enhanced flexibility in drug delivery strategies, capability for content loading, and potential for surface modification [248]. Emerging evidence indicates that MSC-derived EVs exert therapeutic effects through diverse mechanisms.

In AD, MSC-derived EVs facilitate the degradation of Aβ protein and upregulate the expression of genes related to synaptic function, consequently affecting brain glucose metabolism and reversing cognitive dysfunction in AD transgenic mice [249]. Neprilysin (NEP) is an enzyme involved in Aβ degradation, decreasing intracellular Aβ levels. Recent studies have indicated the presence of NEP in MSC-derived EVs derived from adipose tissue, and encapsulating NEP within EVs reduces Aβ production in neuroblastoma cells [250, 251]. Likewise, MSC-derived EVs are capable of suppressing pro-inflammatory factors while enhancing the expression of anti-inflammatory factors, consequently mitigating brain inflammation and exerting neuroprotective effects [252, 253]. Moreover, miR-223-enriched EVs generated from MSCs suppress neuronal apoptosis and promote cell migration by targeting the PTEN and PI3K-Akt pathways [254], suggesting the therapeutic potential of MSC-derived EVs for AD. A recent study suggests that the bone marrow MSC-derived EVs partially rescue dopaminergic neurodegeneration in a *Caenorhabditis elegans* model of PD by diminishing α-synuclein aggregates [255]. In rats injected with 6-hydroxydopamine, administration of EVs derived from MSCs decreased dopaminergic neuron apoptosis and increased dopamine levels in the striatum [256]. Additionally, researchers demonstrated that adipose tissue MSC-derived EVs contain 189 proteins that facilitate cell adhesion and negatively regulate apoptotic pathways. In an in vitro ALS model, these EVs altered apoptotic cascade enzyme expression and increased neuronal activity [257]. Intravenous and intranasal delivery of MSC-derived EVs from adipose tissue protected lumbar spinal cord motor neurons by suppressing glial cell activity in the SOD1^G93A^ mouse model of ALS [258]. In summary, MSC-derived EVs have demonstrated the ability to ameliorate disease phenotypes via multiple mechanisms. Nevertheless, the efficacy of MSC-derived EVs necessitates validation in clinical trials, currently ongoing in China and numerous other nations.

### Neural stem cell (NSC)-derived EVs

NSCs play a crucial role in the nervous system by differentiating into neurons and glial cells [259]. Studies showed that NSC-derived EVs exhibit superior efficacy compared to those from MSCs in improving neurological recovery [260]. This implies that NSC-derived EVs inherit great neurogenic and neuroregenerative potential from their parent cells, rendering them a promising therapeutic option for NDDs.

To date, studies of the therapeutic potential of NSC-derived EVs for NDDs have primarily focused on AD. NSC-derived EVs have shown capacities to ameliorate cognitive impairments in various animal models of AD [261, 262]. Similar to MSC-derived EVs, NSC-derived EVs can also alleviate the accumulation of pivotal pathological molecules in NDDs. Administration of human NSC-derived EVs via retroorbital vein injection markedly attenuated Aβ deposition in the brains of 5 × FAD transgenic mice [262]. NSC-derived EVs exert the treatment effects via immunomodulatory and neuroprotective mechanisms. Following intravenous administration, NSC-derived EVs suppress microglial activation and upregulation of pro-inflammatory cytokines in the brains of AD mice, presumably mediated by the transmission of miR-124 and other inflammation-regulating miRNAs [262]. However, despite the excellent therapeutic efficacy of NSC-derived EVs, the acquisition of NSCs raises ethical concerns, which restrict large-scale EV production [263]. Recent progress in cell programming technology enables the direct conversion of fibroblasts and astrocytes into inducible neural stem cells (iNSCs) possessing robust proliferation and pluripotent differentiation capacities [264]. Intravenous injection of iNSC-derived EVs facilitates neurological function recovery in mouse models of stroke and AD [265, 266]. Hence, both NSC- and iNSC-derived EVs exhibit promising therapeutic effects in both cellular and animal models of NDDs, underscoring the equal importance of assessing the clinical applicability of NSC- and iNSC-derived EVs alongside MSC-derived EVs.

### EVs from other sources

Dendritic cell (DC)-derived EVs have also exhibited therapeutic potential, targeting diverse pathologies due to their immunomodulatory properties. Intravenous administration of rabies virus glycoprotein (RVG)-modified DC-derived EVs loaded with therapeutic BACE1-targeting siRNA in mice inhibited BACE1 expression in the brain, a potential therapeutic target for AD [14]. EVs derived from macrophages have demonstrated efficient BBB penetration and effective delivery of protein cargoes [267]. Intranasal delivery of macrophage-derived EVs containing catalase exhibited neuroprotective effects in PD mouse models [268]. Blood-derived EVs have also been explored as potential therapeutic agents. Intravenously injected blood-derived EVs loaded with dopamine demonstrates brain penetration and high efficacy in PD mouse models, with lower toxicity compared to free dopamine [269]. While various cell types have been used to produce EVs for treating NDDs, more studies are required to understand the potential mechanisms underlying the therapeutic efficacy of stem cell-derived EVs. Concurrently, efforts should be made to comprehensively characterize stem cell-derived EVs to establish standardized clinical protocols, facilitating the translation of EV-based therapies into clinical practice.

### Engineered EVs

Autologous use of EVs reduces immunogenicity, thereby minimizing potential side effects. EVs are ideal carriers, which facilitate cargo delivery while preventing degradation. These attributes have spurred the development of techniques for loading therapeutic cargoes into EVs to address diverse diseases. Cargo loading into EVs can be achieved by manipulating primary cells for cargo overexpression, electroporation of loaded RNAs, or chemical treatment. Plasma-derived EVs may be engineered to carry treatment cargoes. For instance, EVs loaded with dopamine by a saturated solution incubation method can transport dopamine across the BBB to the CNS via transferrin-transferrin receptor interaction [269]. Larger nucleic acids can be loaded via electroporation, with hundreds of molecules per vesicle [270]. However, electroporation-induced nucleic acid aggregation is another challenge, potentially diminishing cargo uptake efficiency and effectiveness [271, 272]. Another strategy is to manipulate parent cells for loading cargoes into EVs through the EV biogenesis pathway. Overexpression of miRNAs using mimics is a prevalent strategy resulting in increased secretion of exogenous miRNAs within EVs. For instance, bone marrow NSCs transfected with miR-146a secreted EVs containing higher levels of miR-146a, and these EVs target the NF-κB pathway, restoring astrocyte activation and ultimately improving cognitive function in AD mice [273]. In addition, administration of adipose-derived MSC EVs loaded with miR-22 enhanced the mobility and memory by suppressing inflammatory factors and reducing pyroptosis in AD model mice [274]. Furthermore, EVs can be loaded with small-molecule drugs for targeted therapy. Montelukast and bryostatin-1, drugs used to treat MS, were encapsulated in NSC-derived EVs, and this EV-mediated drug delivery protected myelin and facilitated remyelination in MS mouse models [275, 276]. This indicates that EVs represent a novel drug delivery platform with significant potential for treating NDDs.

Nanotechnology has been employed to achieve tissue-specific targeting of EVs, enabling precise directional control. Fusion of the CNS-specific RVG peptide with the EV membrane protein Lamp2b enables the construction of a plasmid for producing brain-targeted EVs, facilitating the delivery of desired molecules to the CNS [277]. RVG may enhance exosomal penetration of the BBB by directly interacting with nicotinic acetylcholine receptors expressed on endothelial cells [278, 279]. Numerous studies have employed this strategy to engineer DC- and MSC-derived EVs for delivering siRNAs, shRNAs, and miRNAs to the brain, resulting in significant alleviation of AD and PD phenotypes in vivo [273, 280]. An alternative approach involves expressing the cyclic peptide [c(RGDyK)] on the EV surface, which exhibits a high affinity for integrin αvβ3 on reactive brain endothelial cells [281]. In an in vivo ischemia model, this modification facilitated the preferential accumulation of engineered EVs at the lesion site in the brain compared to unaffected tissue on the contralateral side [281]. Additionally, to address the issue of reduced Aβ clearance efficiency resulting from abnormal microglial lysosomal function in AD, mannose was biologically added to the EV surface to selectively conjugate with the mannose receptor enriched on microglia, thereby targeting these cells [282]. Through this approach, EVs delivered gemfibrozil to enhance the lysosomal activity in microglia, thereby accelerating lysosome-mediated Aβ clearance and effectively improving learning and memory abilities in AD mice [282]. Similarly, engineered EVs expressing PDGFA demonstrated a strong affinity for the OPC surface receptor PDGFRα [275]. Consequently, PDGFA-expressing EVs deliver montelukast to OPCs, promoting oligodendrogenesis and myelin regeneration, and thus ameliorating the phenotype of cuprizone-induced MS models [275].

In summary, EVs have the unique ability to cross the BBB, making them a promising tool for brain-targeting therapies. Compared to synthetic and lipid nanoparticles, EVs exhibit lower immunogenicity, thereby reducing the risk of clinical pseudoallergy [283, 284]. Due to their structural similarity to liposomes and their inherent ability to efficiently transport biomolecules to target cells, EVs present a versatile and potentially superior drug delivery platform [19]. While nanotechnology has advanced clinical drug delivery, the in vivo delivery efficiency of nanoparticles remains low, with most particles rapidly cleared by liver macrophages, limiting their targeted delivery [285]. Although macrophage uptake can be reduced by PEG modification, this limitation has led researchers to explore other EV strategies that can evade immune response. In addition to outperforming synthetic carriers in biodistribution, EV-based therapies uniquely utilize cellular processes for drug loading and surface modification. Genetic engineering enables cells to produce EVs that package therapeutic proteins, RNA molecules, and targeting ligands [286]. Various studies have summarized the administration routes of EVs from different sources for the treatment of neurological diseases, including intranasal administration, intravenous injection, stereotaxic brain injection, and intraventricular injection. The therapeutic effects observed in neurodegenerative animal models are remarkable [258, 287–289]. In another study, Dong et al. employed neutrophil membrane-derived vesicles containing resolvin D2 as an anti-inflammatory agent, specifically delivering it to stroke lesions, which alleviated reperfusion-induced neuroinflammation [290]. Additionally, MSC-derived EVs were administered to patients with steroid-resistant graft-versus-host disease (GvHD) through intravenous infusion every 2 to 3 days over a two-week period. The treatment was well tolerated, with no observed side effects. Notably, GvHD symptoms significantly improved during and after MSC-derived EV treatment, with patients remaining stable for over four months post-treatment [246]. These findings demonstrate the strong safety of EVs as therapeutic agents and their potential for drug delivery. However, challenges remain for EV-based drug delivery, including issues related to EV isolation, characterization, and liver clearance [291]. Overcoming challenges in scalability and reproducibility is crucial for broader application of EV-based therapie.

## Clinical use of EV therapeutic drugs and challenges

EV therapies have shown significant potential in various medical applications. Although it has not yet received formal approval for clinical use, early clinical trials of EV-based therapies have demonstrated certain advantages [292–294]. However, the precise mechanisms underlying the therapeutic effects of EVs remain unclear. Moreover, the standardization of EV extraction protocols, the need of high purity of EV preparations, and the potential side effects of long-term administration, complicate the reproducibility of experimental results and clinical applications. While studies have indicated that EVs exhibit lower immunogenicity than other drug carriers, their potential toxicity in large animal studies and clinical trials is still not fully understood. Early clinical trials have primarily focused on evaluating EV efficacy, but a comprehensive understanding of their potential toxicity and specific mechanisms is equally important for clinical advancement of EV therapies. Since therapeutic EVs are typically derived from human cell lines, they must be rigorously tested in small and large animal models before progressing to human clinical trials. As with other biological therapies, it is crucial to thoroughly assess the immunogenic potential of EVs. Previous research has demonstrated that the immunogenicity and toxicity of EVs are influenced by both their source and the experimental animal model used [295]. Currently, the HEK293T human embryonic kidney cell line is commonly used in EV research due to its capacity for high EV production and efficient genetic modification [295]. Despite these advancements, our understanding of EV immunogenicity in clinical trials remains limited, underscoring the need for further investigation.

Native EVs have inherent limitations, including difficulties in maintaining parental cell culture with minimal metabolic/phenotypic variation over extended periods [296, 297]. To address these issues, novel strategies are being developed to synthesize customized mimetic nanovesicles, such as synthetic EVs and hybrid EVs [298, 299]. Unlike cell-derived EVs, synthetic EVs lack targeting and recognition molecules, necessitating functionalization techniques like bioconjugation and cargo loading to achieve specific therapeutics [300]. Hybrid EVs can be produced by fusing synthetic EVs with native EVs, and these hybrids have the potential to surpass both native EVs and liposomes, offering enhanced targeting capabilities and improved loading stability [301, 302]. As a result, hybrid EVs represent a promising alternative that combines the advantages of native and synthetic systems for therapeutic and diagnostic applications. Addressing key challenges—such as standardized production protocols, ensuring EV purity, monitoring potency, stable targeting, and meeting regulatory requirements—is critical for successful clinical translation [303]. Additionally, current separation techniques for EVs need to be standardized, while concentrating EVs from biological fluids is also challenging due to the low abundance and specificity. Further complexities arise when selecting the optimal route of administration, which must consider factors such as biodistribution, tissue targeting, and immune clearance [304]. Moreover, strict control over the storage and transportation conditions of EVs is essential to preserve their stability and integrity, directly impacting their therapeutic efficacy.

Delivering effective EV-based therapeutics faces several challenges, particularly in determining appropriate dosing strategies to maximize therapeutic benefits while minimizing adverse effects. Key considerations include selecting the correct dose, evaluating efficacy, and determining optimal dosing parameters, such as route, frequency, and timing. To ensure the reliability of EV therapeutics, robust potency assays capable of accurately measuring therapeutic effects in vitro and in vivo are urgently needed [305]. However, the absence of standardized potency assays has hindered their broader acceptance and application. Before EV-based therapeutics can be approved for clinical use, comprehensive analytical evaluations and rigorous safety and efficacy testing are essential. Despite advances in analytical techniques, standardized methods for quantifying EV concentrations and determining appropriate doses are still lacking. Moreover, the efficacy data of EVs for neurological diseases often vary across different dosing regimens and routes of administration, complicating comparisons of therapeutic efficacy even for the same disease. Studies are needed to establish standardized treatment protocols and understand the long-term effects of chronic administration, both of which are critical for clinical translation.

## Conclusion and perspectives

Multiple studies have highlighted the potential use of EVs as biomarkers for diagnosing and assessing cellular and molecular changes in diverse neurological disorders. The successful isolation of distinct subpopulations of BDEs from biological fluids provides a basis for predicting the pathological status of brain cells. In addition, isolation of BDEs from biological fluids is less invasive and more cost-effective. This facilitates the tracking of disease progression and treatment response through repeated measurements. Longitudinal analysis of contents in BDEs can elucidate molecular mechanisms specific to different brain cells in NDDs. Additionally, at initial response stage of cells, BDE vectors correlate with molecular alterations in response to treatment or intervention. Numerous evidence-based laboratory studies have bolstered the clinical potential of BDEs in various neurological diseases, further advancing EV-based diagnostic and therapeutic methodologies for patients.

Furthermore, methods of EV isolation and characterization, as well as standardization of clinically applicable EV preparations, present a considerable challenge. Traditional isolation techniques, such as ultracentrifugation, have low throughput. Emerging techniques like size-exclusion chromatography offer promise for efficient preparation of EVs. However, scaling up cell cultures to generate adequate quantities of EVs for clinical use poses a significant technical challenge. Full characterization of the isolated product is required. Standardization of EVs in formulations is essential, especially for defining their functional activities. Mechanistic studies of specific EV formulations will facilitate the development of suitable dosing strategies and functional evaluations. These will advance our understanding of EV heterogeneity and improve isolation protocols to enrich for vesicles with maximal functional activity.

Another challenge is the development of assays measuring the efficacy of specific populations of EVs. Imparting specific bioactivities to EVs as markers of therapeutic efficacy represents one strategy to tackle this challenge. Establishing such assays holds critical importance for obtaining regulatory approval for EV therapeutics [306]. A crucial question that warrants exploration regarding the utilization of EVs as drug delivery vehicles is whether loading exogenous cargoes will interfere with or interact with endogenous cargoes, potentially leading to off-target effects. The heterogeneity of EVs may pose a significant obstacle if off-target effects surpass therapeutic effects. For instance, delivering a single miRNA within EVs is unlikely to fully address the disease pathology, and a cluster of miRNAs may be necessary to ensure the regulation of both downstream and upstream targets in recipient cells.

Another aspect that requires further investigation is the targeting of EVs to the specific sites where they exert their therapeutic effects. An improved understanding of EV delivery mechanisms is crucial for achieving site-specific targeting, with minimal off-target effects; targeting the therapeutic load to the desired site remains a pivotal issue in designing site-specific EVs. Currently, engineered EVs facilitate drug delivery through the BBB [307], enhancing nutritional support for cells. However, questions remain regarding the dosage of therapeutic vesicles. Recent progress includes the use of small antibody fragments (nanobodies) or RNA aptamers externally attached to EVs, which have exhibited some degree of specificity in targeting therapeutic EVs to designated sites [308].

In summary, BDEs are promising valuable and practical tools that facilitate development of biomarkers for various NDDs. To fully realize this potential, the basic characteristics and complexity of EVs must be precisely defined and controlled. Larger prospective studies should be carefully planned to use standardized procedures for collection, isolation, and characterization of EVs, and reliable data analysis techniques. BDEs offer the potential to use liquid biopsy samples to complement existing clinical biomarkers and neuroimaging modalities, thereby enhancing the diagnosis and prognosis of neurological disorders.

## Data Availability

Not applicable.

## Abbreviations

AD: Alzheimer’s disease
ADEs: Astrocyte-derived EVs
APP: Amyloid precursor protein
BBB: Blood–brain barrier
BDEs: Brain cell-derived extracellular vesicles
BACE1: Beta-secretase 1
CNS: Central Nervous System
CSF: Cerebrospinal fluid
DLP1: Dynamin-like protein 1
EVs: Extracellular vesicles
EBV: Epstein–Barr virus
EDEs: Endothelial cell-derived EVs
ESCRT: Endosomal sorting complex required for transport
ELISA: Enzyme-linked immunosorbent assay
GluR2/3: α-Amino-3-hydroxy-5-methyl-4-isoxazolepropionic acid
GLAST: Glutamine aspartate transporter
GO: Gene Ontology
GvHD: Graft-versus-host disease
HMGB1: High mobility group box 1 protein
HD: Huntington’s disease
HTT: Huntingtin
ILVs: Intraluminal vesicles
ICAM3: Intercellular adhesion molecule 3
IL-6: Interleukin-6
iNSCs: Inducible neural stem cells
KEGG: Kyoto Encyclopedia of Genes and Genomes
L1CAM: Anti-L1 cell adhesion molecule
LPS: Lipopolysaccharide
MVBs: Multivesicular bodies
MDEs: Microglia-derived EVs
miRNA: MicroRNA
MOG: Myelin oligodendrocyte glycoprotein
MSA: Multiple system atrophy
MHC: Major histocompatibility complex
mHTT: Mutated HTT
mtDNA: Mitochondrial DNA
MSCs: Mesenchymal stem cells
MS: Multiple sclerosis
MAP2: Microtubule-associated protein 2
MDVs: Mitochondrial-derived vesicles
mtRNAs: Mitochondrial RNAs
NDDs: Neurodegenerative diseases
NDEs: Neuron-derived EVs
nSMase 2: Neutral sphingomyelinase
NSC: Neural stem cell
NEP: Neprilysin
ODEs: Oligodendrocyte-derived EVs
OPC: Oligodendrocyte progenitor cell
PD: Parkinson’s disease
rRNA: Ribosomal RNA
SNAP25: Synaptosome-associated protein 25
SOD1: Superoxide dismutase 1
RVG: Rabies virus glycoprotein
TMEM119: Transmembrane protein 119
TDP-43: TAR DNA-binding protein-43
